# Investigating Catheter-Related Infections in Southern Benin Hospitals: Identification, Susceptibility, and Resistance Genes of Involved Bacterial Strains

**DOI:** 10.3390/microorganisms11030617

**Published:** 2023-02-28

**Authors:** Victorien Tamègnon Dougnon, Kevin Sintondji, Charles Hornel Koudokpon, Morènikè Houéto, Alidehou Jerrold Agbankpé, Phénix Assogba, Alida Oussou, Anderson Gnamy, Boris Legba, Abdoulaye Idrissou, Honoré Sourou Bankole

**Affiliations:** Research Unit in Applied Microbiology and Pharmacology of Natural Substances, Research Laboratory in Applied Biology, Polytechnic School of Abomey-Calavi, University of Abomey-Calavi, Cotonou BP 526, Benin

**Keywords:** catheter-related infections, venous catheterization, bladder catheterization, antibiotic resistance genes, bacterial virulence genes, southern Benin

## Abstract

The use of catheters and bladder catheters in hospitals can increase the risk of bacterial infections. This study aimed to identify the bacterial strains involved in catheter-related infections (CRI) in southern Benin hospitals. The study included 407 samples, including 95 catheter tip samples and 312 urine samples collected from bladder catheters from patients on the first day and 48 h after admission. The catheter tip samples were analyzed using traditional bacterial isolation and identification methods, while the urine samples were analyzed using VITEK-2. Antibiotic sensitivity was tested using the Kirby Bauer method, and virulence and resistance genes were detected through standard PCR. The results showed a predominance of *Escherichia coli* (53.5%), *Klebsiella pneumoniae* (23.3%), and *Enterobacter aerogenes* (7.0%) among Gram-negative bacilli, and coagulase-negative *Staphylococcus* as the most identified cocci. Bacterial susceptibility to antibiotics showed variable levels of resistance, with *bla*_TEM_ being detected in 42.9% of identified bacterial species, followed by *bla*SHV (26.2%) and *bla*_CTX-M-15_ (16.7%). The *bla*_NDM_ gene was only found in three identified bacterial strains, while *van*A and *van*B genes were detected in 3.2% of strains with a prevalence of 55% for the *mec*A gene. A prevalence of 18.8% for *fim*H was noted for the virulence genes. In conclusion, this study highlights the importance of following proper hygiene and aseptic practices during catheterization to effectively prevent CRIs. These findings should be used to improve interventions in hospitals and reduce healthcare-associated infections in developing countries.

## 1. Introduction

Healthcare-associated infections (HAIs) are a significant concern worldwide, caused by a variety of factors such as poor hospital environments, and the use of invasive devices such as catheters and probes. CRIs are one of the most common HAIs and can lead to increased morbidity, mortality, longer hospital stays, and increased costs [1,2,3,4]. Catheterization is a common procedure in hospital settings, with up to 80% of patients in critical care, neonatology, and pediatrics being catheterized [5]. The use of catheters and bladder catheters can lead to the spread of bacteria and cause infections such as urinary tract infections [6]. Germs can also be responsible for infections known as urinary tract infections related to bladder catheterization [7]. Catheter-related infections (CRIs) are nosocomial infections of concern. They are responsible for increased morbidity and mortality, longer lengths of stay and increased hospital costs [6,8]. These are the most common in the world and can rapidly become complicated with bacteraemia [9]. A total of 12–16% of hospitalized patients will require a urinary examination [10]. The infections often involve bacteria that make therapy complex [11]. Some studies carried out in West Africa have pointed to the real problem posed by infections due to catheters and bladder probes. The prevalence of infections due to catheterization and bladder probes varies from 5.8% to 38.6% in African countries [10,11]. Particularly in Benin, a study by Dougnon and his collaborators carried out in 2016 in the Zinvié’s area hospital (La Croix) showed that only 48 h after a catheter placement, 23.3% of patients (14 out of 60) contracted a urinary tract infection due to bladder catheters. These studies also revealed that the majority of bacteria isolated from catheters and bladder catheters are resistant to ampicillin and cefotaxime. While data and indicators are well monitored in industrialized countries, they are much less so in developing countries like Benin. In addition, the invasive procedures performed by the nursing staff, combined with their poor training, contribute to the alarming situation of these infections [12]. Studies in Benin have revealed poor hygiene in health facilities [13,14,15] and a lack of training of medical personnel in invasive procedures. To address this issue, various intervention projects in Benin aim to improve the technical skills of medical personnel [16]. However, there is no specific study that has analyzed the impact of these factors on the occurrence of infections. In the hospital environment in Benin, there is a risk of nosocomial infections, especially those related to catheterization. To better manage these infections, it is important to understand the bacterial ecology and antibiotic resistance levels involved. The incidence of catheter-related infections is still poorly understood, with only a study by Dougnon et al. [17] showing a high incidence of 33.7% in an urban environment. These observations suggest that the situation may be even more serious in rural areas due to a lack of hygiene and information on preventive measures. Therefore, this study was initiated with the aim of evaluating the status of catheterization and characterizing the bacterial strains involved in catheter-related infections in hospitals in southern Benin. The study aims to answer two questions: What is the level of knowledge about catheterization-related infections in the hospital environment in Benin, and what is the microbiological profile (resistance and virulence) of the bacteria involved in these infections? This study was conducted to assess the situation of catheterization and identify the bacterial strains responsible for CRI in some hospitals located in Southern Benin.

## 2. Materials and Methods

### 2.1. Ethical Approval

The research in our study, including sampling from hospitalized patients, sample processing, and data analysis, was carried out in accordance with the Declaration of Helsinki and was approved by the National Health Research Ethics Committee of Benin under reference number N°67/MS/DC/SGM/DRFMT/CNERS/SA dated 20 April 2021. Furthermore, written informed consent was obtained from all patients and/or their legal representatives.

### 2.2. Study Design

This study was a prospective and descriptive study conducted in southern Benin between June 2021 and June 2022. The study was carried out in two stages. The first stage took place from June to August 2021 in the Departmental Hospital of Lokossa and the Comè’s Area Hospital, located in rural areas. The study population consisted of neonates, children, and adults who underwent catheterization during the study period. Patients who were unable to undergo catheterization and those who were diagnosed with sepsis before catheterization were excluded. The catheter tips were collected from the veins of the patients. The techniques and methods used by health workers involved in catheterization were recorded, and the practice of hygiene measures was evaluated. A compliance rate of at least 60% was considered acceptable. The second stage of the study was conducted in four hospitals in Cotonou, including the Hubert Koutoukou Maga’s National University Hospital Center, the Bethesda Health Center, Saint Luc’s Hospital, and Suru-Léré’s Hospital. The emergency, medical, gynecological, and surgical departments were included in the study. Urine was collected from patients who underwent bladder catheterization, along with socio-demographic data. All patients who consented, either verbally or through their parents in the case of comatose patients, were included in the study. The study was approved by the National Health Research Ethics Committee of Benin (N°67/MS/DC/SGM/DRFMT/CNERS/SA of 20th April 2021) and all patients or their legal representatives provided written informed consent.

### 2.3. Samples Collection

The sample size was determined using the Schwartz [18] formula $n=\frac{z^{2}\times p\times q}{d^{2}}$, with *n* = required sample size, *p* = prevalence; *p* = 0.5; q = 1 − *p*; z = confidence level according to the centered reduced normal distribution (for a confidence level of 95%, z = 1.96); d = margin of error tolerated for this study equal to 0.05 [18]. So, *n* = 385.

Thus, we collected a total of 407 samples, including 95 catheter tips and 312 urine samples. Of the 95 catheter samples, 30 were from the Departmental Hospital of Lokossa and 65 from the Comè Zone Hospital. The 312 urine samples from bladder catheters were collected from 85 patients at the CNHU of Cotonou, 70 patients at the Bethesda Health Center, 80 patients at Saint Luc Hospital, and 77 patients at Suru-Léré’s Hospital. The catheter tips were immediately collected after use and placed in sterile, dry tubes. The urine samples were collected 10 minutes after the catheterization and 48 h later (Day 3) using a 10 mL single-use syringe. The catheter was clamped for 10 min to allow urine to accumulate upstream before being disinfected with alcohol. The samples were then transported in coolers with accumulators to the Laboratory of the Research Unit in Applied Microbiology and Pharmacology of Natural Substances at the University of Abomey-Calavi for analysis.

### 2.4. Isolation and Identification of Bacterial Strains

For each catheter sample, 3 mL of Mueller Hinton broth was added, and it was then incubated at 37 °C for 24 h. Every 24 h, the culture broth was then inoculated on Eosin Methylene Blue (EMB), mannitol salt, Mueller Hinton (MH) agar enriched with fresh sheep blood, and incubated again at 37 °C for another 24 h. The isolated colonies underwent Gram staining and purification, and a Gram control was performed to verify the purity of the strains. The catalase test, free staphylocoagulase test, DNase test, and the determination of the type of hemolysis for catalase-negative strains were carried out to identify Gram-positive cocci. The identification of Enterobacterales species was done using API 20 E gallery.

### 2.5. Antibiotic Susceptibility Testing of Identified Species

The susceptibility of the isolated Enterobacterales was tested against 13 different antibiotics using the disc diffusion method on Mueller Hinton agar medium. This was done in accordance with the recommendations of the Antibiogram Committee of the French Society of Microbiology [19]. The bacterial suspension was standardized using the McFarland 0.5 control, and the following antibiotics were studied: Amoxicillin + clavulanic acid (30 µg), Ampicillin (10 µg), Fosfomycin (30 µg), Imipenem (10 µg), Ceftriaxone (30 µg), Nalidixic acid (30 µg), Oxacillin (5 µg), Chloramphenicol (25 µg), Vancomycin (5 µg), Erythromycin (15 µg), Kanamycin (30 µg), Gentamycin (10 µg), and Tobramycin (10 µg).

The analysis of urine samples was performed to identify bacterial species and determine their antibiotic susceptibility profile using the VITEK 2 COMPACT-AST-N233 system according to the manufacturer’s recommendations from BioMerieux^®^. The card used for the antibiotic susceptibility test contained the following antibiotics: Ampicillin (10 µg), Amoxicillin (20 µg), Amoxicillin/Clavulanic acid (20–10 µg), Cefalotin (30 µg), Cefotaxime (5 µg), Cefoxitin (30 µg), Ceftaxidime (10 µg), Ciprofloxacin (5 µg), Gentamicin (10 µg), Imipenem (10 µg), Nalidixic Acid (30 µg), Ofloxacin (5 µg), Ertapenem (10 µg), Nitrofurantoin (100 µg), Piperacillin/Tazobactam (30–6 µg), Ticarcillin (75 µg), Tobramycin (10 µg), and Trimethoprim-sulfamethoxazole (1.25–23.75 µg).

### 2.6. Molecular Identification of Resistance and Virulence Genes

The genetic material (DNA) of the identified bacterial species was extracted and the standard PCR technique was used to detect resistance genes (*bla*_CTXM-1_, *bla*_CTXM-15_, *bla*_SHV_, *bla*_TEM_, *bla*_NDM_, *bla*_VIM_, bla_KPC_, and bla_OXA-48_ for enterobacteria strains, and *van*A, *van*B, and *mec*A for Gram-positive cocci strains) and virulence genes (*fim*H and ISS). The PCR protocol consisted of an initial denaturation step at 94 °C for 4 min for all the genes, followed by specific cycles for each gene. For *bla*_CTXM-1_, *bla*_CTXM-15_, *bla*_SHV_, *bla*_TEM_, *bla*_NDM_, *bla*_VIM_, *bla*_KPC_, and *bla*_OXA-48_ genes, 30 cycles of denaturation at 94 °C for 30 s, hybridization at 55 °C for 30 s, and elongation at 72 °C for 40 s were performed. For *mec*A, *van*A, and *van*B genes, 30 cycles of denaturation at 94 °C for 60 s, hybridization at 50 °C for 60 s, and elongation at 72 °C for 60 s were performed. Finally, for *fim*H and ISS genes, 40 cycles of denaturation at 94 °C for 40 s, hybridization at 50 °C for 60 s, and elongation at 72 °C for 60 s were performed. The cycles were followed by a final elongation step at 72 °C for 5 min. The primers used in the PCR reaction are listed in Table 1.

### 2.7. Data Analysis

The statistical analysis was conducted using IBM SPSS Statistics 22 software. The graphs were created and edited using GraphPad Prism. The Chi^2^ test was employed to determine any possible associations between catheter-associated infections and the following variables: age, gender, appearance of bandages during catheter removal, type of product infused through the catheter, use of antiseptic prior to catheter placement, use of antiseptic during catheter placement, type of antiseptic solution used, duration of catheterization, presence of varnish after catheterization, hand hygiene practices before and during catheter removal, sterilization of materials, size of catheter, type of vein, placement site, and presence of tubular reflux during catheter removal.

## 3. Results

### 3.1. Venous Catheter Infection

A total of 95 catheter tips was collected for this study. Among the samples collected, 41 were positive after bacterial culture.

#### 3.1.1. Catheterization in the Departmental Hospital of Lokossa and the Comè Zone Hospital

The results of the study showed that the majority of the samples (68.4%) came from Comè’s hospital, with a higher percentage of female patients (62.1%) compared to male patients (37.9%). The infection due to catheterization was more prevalent in women (33.7%). The age groups of 0-10 years and 20-30 years were the most represented (26.3% and 25.3% respectively). Nurses were the most represented professional category (78.9%). The most commonly used catheter sizes were G20 and G24 (45.3% and 27.4% respectively) and the peripheral vein was the most used (97.8%) with the upper limb being the most commonly used placement site (93.7%). In 52% of the samples, the duration of catheterization was between 48 and 96 h. Antiseptic was used before catheter insertion in 98.95% of cases, but during removal in only 78.9% of cases. Hand washing was performed in 74.7% of cases, but the material used during catheterization was not sterile in 94.7% of cases. There was no tubular reflux during catheter removal in 75.8% of cases, and germs were found in 43.2% of catheters. Alcohol at 70° was the most commonly used solution for disinfection (91.6%).

The study showed a significant association between the aspect of the bandages during removal and the infection of the catheter (*p* = 0.013), with 47.4% of the bandages being soiled. The study also showed a significant link between the nature of the product injected by the catheter and the infection of the catheter (*p* = 0.028). The bacterial culture revealed that 14.6% of the samples were polymicrobial (Table 2).

#### 3.1.2. Identified Bacterial Species and Their Resistance Profile

**Figure 1:**
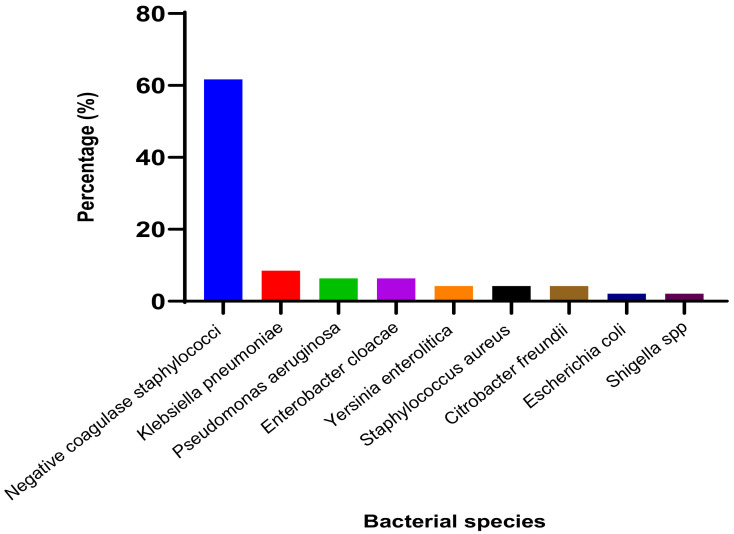
Percentage of bacterial species isolated from catheter tip samples.

The results of the catheter bacterial culture showed that 14.63% of the samples were found to contain multiple types of bacteria. The most common species were Coagulase Negative *Staphylococci*, accounting for 61.70%, followed by *Klebsiella pneumoniae* (8.5%), *Pseudomonas aeruginosa* (6.4%), and *Enterobacter cloacae* (6.4%) as shown in Figure 1. The results of the antibiotic susceptibility test indicated that coagulase negative *Staphylococci* displayed strong resistance to chloramphenicol (54.8%) and nalidixic acid (67.7%). The gram-negative bacilli showed resistance to ceftriaxone (68.7%) and ampicillin (75%). However, no resistance was observed for imipenem (Table 3 and Table 4).

#### 3.1.3. Resistance and Virulence Genes Detected

The results of the study showed that 55% of the *Staphylococci* strains from catheter tips tested positive for the *mec*A gene. In contrast, the *van*A and *van*B genes were found in only 1% of the coagulase-negative *Staphylococci* strains. The *bla*_TEM_ gene was present in 47.8% of the Gram-negative strains. All of the *Enterobacter cloacae* and *Escherichia coli* strains tested positive for the *bla*_TEM_ gene, while none of the *Klebsiella pneumoniae* strains tested positive for the *bla*_TEM_ gene, but all carried the *bla*_SHV_ gene. The *bla*_CTX-M-15_ gene was the most common in both enterobacteria and non-enterobacteria strains, found in 50% of the strains tested. Out of these strains, only 18.8% of *Klebsiella pneumoniae* species tested positive for the *fim*H virulence gene. No strains tested positive for the ISS virulence gene. It is worth noting that, in addition to their resistance to antibiotics, the bacterial species responsible for bacteremia due to catheter-related care are also highly virulent (Table 5)

### 3.2. Bladder Catheter Infections

A total of 312 catheter bladder were collected for this study. Among the samples collected, 86 were positive after bacterial culture.

#### 3.2.1. Sociodemographic Data and Clinical Factors Related to the Occurrence of Urinary Tract Infections in Cotonou Hospitals in Benin

The results of the study showed that 64.7% of the patients who provided urine samples were female. More than 90% of the patients who had a urinary tract infection were under 70 years of age (Table 6). The majority (31%) of the patients treated with third-generation cephalosporins had infections with a risk factor, representing 12.3% of the total cases. Out of the reasons for hospitalization, 4.5% of women who underwent caesarean section contracted a urinary tract infection, while 24.36% of those who had a urinary catheter in place also developed an infection (OR = 0.97) (Table 7).

#### 3.2.2. Identified Bacterial Species and Their Resistance Profile

**Figure 2:**
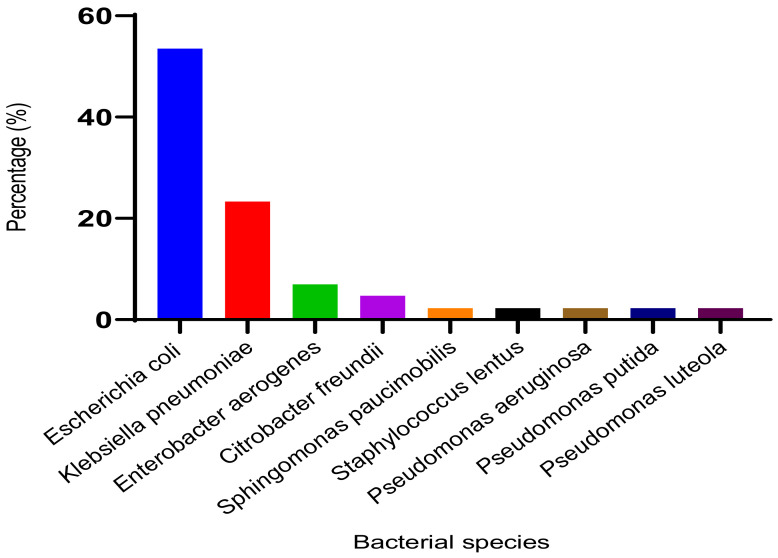
Percentage of bacterial species involved in urinary tract infections.

The most commonly isolated bacterial species from urine samples were *Escherichia coli* (53.5%), *Klebsiella pneumoniae* (23.3%), *Enterobacter aerogenes* (7%) and *Citrobacter freundii* (4.7%) (Figure 2). The different bacterial strains isolated showed varying patterns of antibiotic resistance. The isolated strain of *Staphylococcus lentus* was resistant to all antibiotics except clindamycin and vancomycin. Among the beta-lactam antibiotics tested on the isolated Gram-negative bacilli, the highest rates of resistance were observed for ticarcillin (93.02%), amoxicillin (79.07%), cephalothin (62.07%) and cefotaxime (53.48%). A high resistance rate (86.05%) was also noted for the trimethoprim/sulfamethoxazole combination. Overall, 74.42%, 65.12% and 62.79% of the Gram-negative bacilli were resistant to nalidixic acid, ciprofloxacin, and ofloxacin, respectively. All strains were sensitive to ertapenem. Regarding the isolated Enterobacterales strains, all *Escherichia coli*, *Klebsiella pneumoniae*, and *Enterobacter aerogenes* strains were resistant to ticarcillin, and all Escherichia coli and Klebsiella pneumoniae strains were resistant to amoxicillin. For the non-enterobacterial strains, with the exception of *Pseudomonas aeruginosa* strains, which were only resistant to cefotaxime, the other strains of other species were multidrug-resistant (Table 8).

#### 3.2.3. Resistance and Virulence Genes Detected

A total of five resistance genes was detected in bacterial species from urine samples, including *bla*_TEM_ (42.9%), *bla*_SHV_ (26.2%), *bla*_CTXM1_ (28.6%) and *bla*_CTXM15_ (16.7%). The *bla*_NDM_ gene (7.1%) was the only carbapenem resistance gene detected and was found in *Escherichia coli* and *Klebsiella pneumoniae*.

All detected genes were found in at least one strain of *Escherichia coli* or *Klebsiella pneumoniae*. No resistance genes were detected in *Staphylococcus lentus* (Table 9).

## 4. Discussion

### 4.1. Epidemiological Characteristics of the Study Population and Aspects Related to In-Hospital Care

The impact of catheterization on the occurrence of bacteraemia is of utmost significance in health care. Our study included patients of various genders and ages. The sample size of female patients was larger than that of male patients, and the examination of the relationship between patient gender and catheter infection showed a significant correlation. This suggests that women are more likely to be hospitalized for catheter-related care and are more susceptible to infections. These findings are in line with previous studies, such as [25], where most bladder catheter patients in a southern Benin hospital were female (75%). Many studies worldwide have reported a high incidence of urinary tract infections in female patients with bladder catheters [26,27]. A meta-analysis of various studies on risk factors for catheter-associated urinary tract infections found that female gender was a commonly reported determinant factor [28]. This female predilection may be attributed to anatomy, as the female urethra is short and wide, providing a direct pathway for bacteria to reach the bladder [27]. The age group 0-10 years (26.3%) was the most prevalent among patients from whom catheter tips were collected. This is in line with previous research, which shows that catheter-related bacteraemia is more common in neonates and children [29]. This can be partly attributed to the immune incompetence of children, especially neonates [29]. According to Douard [30], the frequency of catheter-related bacteraemia remained constant throughout the hospitalization period. In this study, 98.9% of nurses utilized good aseptic techniques before catheter placement, although they were unaware of their importance in catheter removal (78.9% of cases). The disinfection process was of good quality and alcohol at 70 °C was the most commonly used solution (91.6%). Handwashing was performed before catheter insertion and during removal in 74.7% of cases. These results indicate that disinfection, the alcohol solution used, or the cleanliness of the hands of the catheterization agents were not related to the causes of bacteraemia. However, the material used during catheterization in the participating hospitals was not sterile in 94.7% of cases, and 47.4% of bandages were soiled. This highlights the need for education and training for healthcare professionals to emphasize the importance of using sterile materials and proper techniques for removing catheters to reduce the risk of infections. Non-sterilized equipment and contaminated bandages can increase the risk of infection in patients after catheter placement. This conclusion aligns with the findings of Dougnon et al. [17], who demonstrated a significant correlation between infection and the condition of the bandages at catheter removal. The relationship between infection and bandage condition may be due to the contamination of the bandages. It is recommended to cover the catheter insertion site with a sterile, transparent polyurethane bandage (B-3) to facilitate monitoring. Previous research has shown that increasing age is a risk factor for urinary tract infections (UTIs) in catheterized patients. Although over 90% of the patients in our study were under 70 years of age, special consideration should be given to elderly patients undergoing catheterization [31].

### 4.2. Bacterial Species Identified and Sensitivity to Antibiotics

The most commonly identified bacterial species found at the end of the catheters were coagulase-negative *Staphylococcus* (61.7%), followed by *Pseudomonas aeruginosa* (6.4%), *Citrobacter freundii* (4.3%), *Enterobacter gergoviae* (2.1%), and *Enterobacter spp*. (4.3%). Other species included *Escherichia vulneris* (2.1%), *Klebsiella oxytoca* (4.3%), *Klebsiella pneumoniae* (2.1%), *Klebsiella spp*. (2.1%), *Shigella sonnei* (2.1%), *Staphylococcus aureus* (4.3%), and *Yersinia pestis* (4.3%). According to Lachassinne et al. [32], Gram-positive cocci, such as *Staphylococcus aureus* and coagulase-negative *Staphylococcus*, are involved in 75% of catheter-related infections. Our results align with these findings. Coagulase-negative *Staphylococci* have been identified as a significant contributor to nosocomial bacteremia in numerous studies [33,34,35]. This highlights the importance of addressing the issue for the overall well-being of the population, especially children. Several studies, including those by Floret et al. [36], have shown the role of *Pseudomonas aeruginosa* in hospital-acquired infections, and our results are consistent with the findings of Lukuke et al. [37]. Our results did not show *Escherichia coli*, *Proteus vulgaris*, or *Enterobacter cloacae*, which was different from the findings of Dougnon et al. [17]. *Yersinia pestis* was not reported in Dougnon et al. [17]. These differences could be attributed to the specific care environment. Antibiotic susceptibility testing showed a high prevalence of multidrug-resistant strains, including resistance to ceftriaxone. This highlights the importance of finding solutions to improve patient care, especially in light of the ongoing shortage of antibiotics. This observed resistance is believed to be due to the frequent use of antibiotics in treating bacterial infections. The antibiogram of the cocci showed that 16.1% were resistant to vancomycin.

The high prevalence of *E. coli* among the Gram-negative bacilli isolated from urine samples supports the findings of Koçak et al. [38], who reported that this bacterium is the most commonly involved in catheter-associated UTIs. This result is also consistent with the results of several studies conducted in other countries that focused on the role of enterobacteria in nosocomial UTIs [39,40,41]. The high involvement of E. coli in UTIs can be attributed to its pathogenic factors, such as pili that enable it to bind to the urinary epithelium and prevent elimination through urine [42]. In addition to *E. coli*, other bacteria were also isolated, including *E. aerogenes* (7%), *Citrobacter freundii* (4.7%), and non-enterobacteria, such as *Pseudomonas luteola*, *P. aeruginosa*, *P. putida*, and *S. paucimobilis* (2.3% each). The role of these bacteria in nosocomial UTIs has been reported in previous studies [43]. Our study also identified the presence of *Staphylococcus lentus*, which is consistent with the findings of Al-azawi et al. [44]. Antibiotic susceptibility testing showed that the *S. lentus* strain was resistant to all antibiotics except clindamycin and vancomycin, similar to the results of Al-Salamy et al. [45].

For Gram-negative bacilli, high resistance to beta-lactams, such as ticarcillin (93%), amoxicillin (79.1%), cefalotin (62.1%), and cefotaxime (53.5%), was observed. This resistance is also reported by [46]. However, all strains were sensitive to ertapenem, as reported by Chaussade et al. [47]. This differential resistance may be due to the overconsumption of antibiotics in developing countries [48] and self-medication, as well as a lack of infection management guidelines [49]. Among the enterobacteria, all strains of *E. coli*, *Klebsiella pneumoniae*, and *E. aerogenes* were resistant to ticarcillin and amoxicillin. Additionally, 100% of *E. aerogenes* and 95.7% of *E. coli* strains were resistant to the combination of trimethoprim/sulfamethoxazole, which is consistent with the findings of Konaré [50]. All *Citrobacter freundii* strains were resistant to amoxicillin/clavulanic acid, cefalotin, cefoxitin, and nalidixic acid, similar to the results of Kbirou et al. [51]. The non-enterobacteria strains were mostly multidrug-resistant, with the exception of *P. aeruginosa* strains that were only resistant to cefotaxime, consistent with the results of Bitsori et al. [52]. The study also revealed a total of five resistance genes.

### 4.3. Resistance and Virulence Genes Detected

The ranking of bacterial species found in urine samples showed that the most frequently detected resistance gene was bla_TEM_ (42.9%), followed by bla_SHV_ (26.2%), bla_CTX-M1_ (28.6%), and bla_CTX-M15_ (16.7%). These results align with the results of phenotypic tests, which showed a high incidence of beta-lactam resistance. Prior studies have also reported the presence of these same resistance genes in uropathogenic bacteria, with bla_CTX-M_ being the most prevalent [53,54]. Bacteria employ various mechanisms to resist antibiotics, one of which is the inactivation of the antibiotic through the production of enzymes such as β-lactamases [55,56]. This mechanism represents a significant factor in beta-lactam resistance [57]. β-lactamases can inactivate penicillins, cephalosporins, monobactams, and carbapenems by breaking down the amide bond in the β-lactam ring [58]. The most commonly found enzymes are TEM, SHV, CTX-M, OXA, VIM, IMP, and NDM variants [59]. These genes are often carried by transmissible plasmids and can be horizontally transferred between species [60]. As a result, bacteria that produce these enzymes become multidrug-resistant, reducing treatment options for infections [61]. The bla_NDM_ gene (7.1%), which confers carbapenem resistance, was the only carbapenem resistance gene found and was present in Escherichia coli and Klebsiella pneumoniae. All of the detected genes were present in at least one strain of E. coli or K. pneumoniae. This finding is consistent with the results of a study by El-Houssaini et al. [62], which showed an increase in the frequency of β-lactamase resistance genes (bla_TEM_, bla_SHV_, and bla_CTX-M_) in isolates of E. coli and K. pneumoniae. The potential transfer of ESBL genes to other bacteria is a significant concern in the clinical management of infections caused by ESBL-carrying bacteria [62].

## 5. Conclusions

This study found a high incidence of Catheter-Related Infections (CRIs) and Urinary Tract Infections (UTIs) associated with bladder catheterization. Factors such as gender, the appearance of the catheter dressing upon removal, the nature of the product injected through the catheter, and the professional category of the nurse who inserts the catheter increase the risk of UTI in patients and are also related to the occurrence of catheter-related bacteremia. The identified bacterial species were multidrug-resistant and carried resistance and virulence genes, constituting a major public health concern. This study will be used to improve patient management in hospitals. However, the main limitation is the absence of sequencing of the multidrug-resistant strains isolated in this study. Further sequencing and analysis of sequences would have provided a deeper understanding of the epidemiology of catheter-related infections in Benin.

## Figures and Tables

**Table 1:** List of genes searched and their different primers.

Genes	Primers	Sequence 5′-3′	References
*bla*_TEM_	TEM F	ATGAGTATTCAACATTTCCGC	[20]
	TEM R	CAATGCTTAATCAGTGAGG	
*bla_SHV_*	SHV F	AAGATCCACTATCGCCAGCAG	
	SHV R	ATTCAGTTCCGTTTCCCAGCGG	
*bla*_CTX-M-1_	CTX-M-1 F	GGTTAAAAAATCACTGCGTC	
	CTX-M-1 R	TTGGTGACGATTTTAGCCGC	
*bla*_CTX-M-15_	CTX-M-15 F	CACACGTGGAATTTAGGGACT	
	CTX-M-15 R	GCCGTCTAAGGCGATAAACA	
*bla*_KPC_	KPC F	CGCCAATTTGTTGCTGAAGG	[21]
	KPC R	CAGGTTCCGGTTTTGTCTCC	
*bla*_NDM_	NDM F	GTTTGATCGTCAGGGATGGC	
	NDM R	CTCATCACGATCATGCTGGC	
*bla*_VIM_	VIM F	GATGGTGTTTGGTCGCATATC	
	VIM R	CGTCATGAAAGTGCGTGGAG	
*bla*_IMP_	IMP F	GAAGGCGTTTATGTTCATAC	
	IMP R	GTACGTTTCAAGAGTGATGC	
*bla*_OXA-48_	OXA-48 F	GGTAGCAAAGGAATGGCAAGAA	
	OXA-48 R	CGACCCACCAGCCAATCTTA	
*van*A	*van*A F	GGGCTGTGAGGTCGGTTG	[22]
	*van*A R	TTCAGTACAATGCGGCCGTTA	
*van*B	*van*B F	TTGTCGGCGAAGTGGATCA	
	*van*B R	AGCCTTTTTCCGGCTCGTT	
*mec*A	*mec*A F	GTTAGATTGGGATCATAGCGTCATT	[23]
	*mec*A R	TGCCTAATCTCATATGTGTTCCTGTAT	
*fim*H	*fim*H F	TACTGCTGATGGGGCTGGTC	[20,24]
	*fim*H R	TACTGCTGATGGGGCTGGTC	
ISS	ISS F	GGCAATGCTTATTACAGGATGTGC	
	ISS R	GAGCAATATACCCGGGGCTTCC	

**Table 2:** Sociodemographic data and some data related to catheterization in the departmental Hospital of Lokossa and the Comè’s Area Hospital.

	Positive	Negative	Total	Chi^2^	Odds Ratio	Z
Sex						
Male	9 (9.5%)	27 (28.4%)	36 (37.9%)	0.005	0.068	−3.53
Female	32 (33.7%)	27 (28.4%)	59 (62.1%)			
Age						
(0–10)	13 (13.7%)	12 (12.6)	25 (26.3%)	0.330	0.91	−0.53
(10–20)	4 (4.2%)	6 (6.3%)	10 (10.5%)			
(20–30)	11 (11.6%)	13 (13.7%)	24 (25.3%)			
(30–40)	2 (2.1%)	11 (11.6%)	13 (13.7%)			
(40–50)	7 (7.4%)	4 (4.2%)	11 (11.6%)			
(50–60)	2 (2.1%)	2 (2.1%)	4 (4.2%)			
(60–70)	1 (1.1%)	3 (3.2%)	4 (4.2%)			
(70–80)	1 (1.1%)	3 (3.2%)	4 (4.2%)			
Professional category						
Nurse	33 (34.7%)	42 (44.2%)	75 (79%)	0.552	0.818	−0.45
Health care aide	1 (1.1%)	4 (4.2%)	5 (5.3%)			
Midwife	7 (7.4%)	8 (8.4%)	15 (15.8			
Catheter size						
G18	5 (5.3%)	11 (11.6%)	16 (16.8%)	0.173	0.348	−239
G20	16 (16.8%)	27 (28.4%)	45 (45.3%)			
G22	7 (7.37%)	3 (3.16%)	10 (10.5%)			
G24	13 (13.7%)	13 (13.7%)	26 (27.4%)			
Type of veins						
Peripheral	40 (42.1%)	53 (55.8%)	93 (97.9%)	0.843	0.15	−1.04
Central	1 (1.1%)	1 (1.1%)	2 (2.1%)			
Placement site						
Upper extremity	39 (41.05%)	50 (52.6%)	89 (93.7%)	0.744	13.83	1.44
Lower limb	1 (1.1%)	3 (3.2%)	4 (4.2%)			
Jugular	1 (1.01%)	1 (1.1%)	2 (2.1%)			
Use of antiseptics before						
Yes	10 (10.5%)	10 (10.5%)	20 (21.1%)	0.487	3.56	1.56
No	31 (32.6%)	44 (46.3%)	75 (79%)			
Used solution						
Alcohol 70	39 (41.1%)	48 (50.5%)	87 (91.6%)	0.396	1.5	0.63
Beta-block alcohol	1 (1.1%)	1 (1.1%)	2 (2.1%)			
Others	1 (1.1%)	5 (5.3%)	6 (6.3%)			
Disinfection quality						
Good	41 (43.2%)	54 (56.8%)	95 (100%)			
Duration of catheterization						
1	1 (1.1%)	0 (0%)	1 (1.1%)	0.492	1.2	0.90
2	7 (7.4%)	9 (9.5%)	16 (16.8%)			
3	15 (15.8%)	13 (13.7%)	28 (29.45%)			
4	10 (10.5%)	14 (14.7%)	24 (25.3%)			
5	6 (6.3%)	12 (122%)	18 (19%)			
6	1 (1.1%)	3 (3.2%)	4 (4.2%)			
7	0 (0%)	2 (2.1%)	2 (2.1%)			
8	0 (0%)	1 (1.1%)	1 (1.1%)			
>8	1 (1.1%)	0 (0%)	1 (1.1%)			
Presence of varnish after catheter placement						
Present	4 (4.2%)	8 (8.4%)	12 (12.6%)	0.462	0.66	−0.51
Absent	37 (39%)	46 (48.4%)	83 (83.4%)			
Hand washing before catheter insertion						
Yes	32 (33.7%)	39 (41.1%)	71 (74.7%)	0.517	1.89	0.90
No	9 (9.5%)	15 (15.8%)	24 (25.3%)			
Appearance of the bandages upon removal						
Dirty	22 (23.2%)	23 (24.2%)	45 (47.4%)	0.0013	1.185	0.73
Unstuck	3 (3.2%)	0 (0%)	3 (3.2%)			
Unstuck and dirty	2 (2.1%)	14 (14.7%)	16 (16.8%)			
Hermeneutical and clean	14 (14.7%)	17 (17.9%)	31 (32.6%)			
Nature of the injected product						
Antibiotic	34 (35.8%)	52 (54.7%)	86 (90.5%)	0.028	0.087	−2.21
Not antibiotic	7 (7.4%)	2 (2.1%)	9 (9.3%)			

**Table 3:** Resistance to antibiotics in strains of Gram-positive cocci.

	AMP	OX	TOB	C	E	NA	FOX	VAN
*S. aureus*	-	1 (50%)	-	2 (100%)	1 (50%)	1 (50%)	-	1 (50%)
*NCS*	5 (17%)	9 (31%)	11 (38%)	15 (52%)	11 (38%)	20 (72%)	4 (14%)	4 (14%)
Total	5 (16.1%)	10 (32.4%)	11 (35.5%)	17 (54.8%)	12 (38.7%)	21 (67.7%)	4 (12.9%)	5 (16.1%)

AMP: Ampicillin OX: Oxacillin, TOB: Tobramycin, C: Chloramphenicol, E: Erythromycin, NA: Nalidixic Acid, FOX: Fosfomycin and VAN: Vancomycin; *S. aureus*: *Staphylococcus aureus;* -: No resistance.

**Table 4:** Resistance to antibiotics in strains of Gram-negative bacilli.

	AMP	AMC	CRO	IMP	KAN	GEN	NA	FOX
*Yersinia enterolitica*	1 (50%)	1 (50%)	1 (50%)	-	1 (50%)	1 (50%)	-	-
*Shigella* spp.	-	1 (100%)	-	-	-	-	1 (100%)	-
*Klebsiella* *pneumoniae*	4 (100%)	4 (100%)	2 (50%)	-	2 (50%)	2 (50%)	2 (50%)	-
*Enterobacter* *cloaceae*	3 (100%)	2 (75%)	3 (100%)	-	3 (100%)	3 (100%)	-	1 (25%)
*Pseudomonas* *aeruginosa*	3 (100%)	1 (33%)	3 (100%)	-	1 (33%)	1 (33%)	1 (33%)	3 (100%)
*Escherichia coli*	1 (100%)	1 (100%)	1 (100%)	-	1 (100%)	1 (100%)	-	-
*Citrobacter freundii*	1 (50%)	-	1 (50%)	-	-	2 (100%)	1 (50%)	-
Total	13 (81.3%)	10 (62.5%)	11 (68.8%)	-	8 (50%)	10 (62.5%)	5 (31.3%)	4 (25%)

AMP: Ampicillin, AMC: Amoxicillin + clavulanic acid, CRO: Ceftriaxone, IMP: Imipenem, KAN: Kanamycin GEN: Gentamycin NA: Nalidixic Acid and FOX: Fosfomycin; -: No resistance.

**Table 5:** Distribution of resistance and virulence genes in enterobacterial and non-enterobacterial strains.

	Resistance Genes			Virulence Genes	
	*bla*_TEM_	*bla*_SHV_	*bla*_CTX-M-15_	*fim*H	ISS
*Yersinia enterolitica*	1 (50%)	1 (50%)	2 (100%)	-	-
*Shigella* spp.	1 (100%)	1 (100%)	-	-	-
*Klebsiella pneumoniae*	-	4 (100%)	1 (25%)	4 (100%)	-
*Enterobacter cloaceae*	3 (100%)	-	3 (100%)	-	-
*Pseudomonas aeruginosa*	-	-	-	-	-
*Escherichia coli*	1 (100%)	-	1 (100%)	-	-
*Citrobacter freundii*	1 (50%)	-	2 (100%)	-	-
Total	7 (54%)	6 (37.5%)	8 (50%)	4 (25%)	-

-: Absence of resistance genes.

**Table 6:** Sociodemographic data.

Variable	Negative	Positive	Total	Chi^2^	OR
Sex					
Female	176 (50%)	46 (14.7%)	202 (64.7%)	0.070	1.72
Male	70 (22.4%)	40 (12.8%)	110 (35.3%)		
Total	226 (72.4%)	86 (27.6%)	312 (100%)		
Age					
(20, 30)	52 (16.7%)	18 (5.7%)	70 (22.4%)	0.103	0.96
(30, 40)	62 (19.87%)	14 (4.5%)	76 (24.4%)		
(40, 50)	38 (12.2%)	16 (5.1%)	54 (17.3%)		
(50, 60)	24 (7.7%)	22 (7.1%)	46 (14.7%)		
(60, 70)	30 (9.62%)	14 (4.50%)	44 (14.1%)		
(70, 80)	18 (5.8%)	0 (0.00%)	18 (5.6%)		
(80, 90)	2 (0.6%)	2 (0.6%)	4 (1.3%)		

**Table 7:** Clinical factors linked to the occurrence of urinary tract infections by catheterization.

Variable	Negative	Positive	Total	Pr Chi^2^	OR
Routine antibiotic therapy					
Yes	110 (35.3%)	56 (18%)	166 (53.2%)	0.066	0.36
No	116 (37.2%)	30 (9.6%)	146 (46.8%)		
Total	226 (72.4%)	86 (27.6%)	312 (100%)		
Type of antibiotic therapy					
C3G + Quinolones	6 (1.9%)	6 (1.9%)	12 (3.9%)	0.145	0.96
3G + SXT	22 (7.1%)	2 (0.6%)	24 (7.7%)		
C3G + Aminosides	4 (1.3%)	2 (0.6%)	6 (1.9%)		
C3G	58 (18.7%)	38 (12.3%)	96 (31%)		
No ATB	122 (38.7%)	32 (10.3%)	154 (49%)		
Aminosides	6 (1.9%)	4 (1.3%)	10 (3.2%)		
Quinolones	2 (0.6%)	2 (0.6%)	4 (1.3%)		
Glycopeptides	6 (1.9%)	0 (0.0%)	6 (1.9%)		
Total	226 (72.4%)	86 (27.6%)	312 (100%)		
Reason for hospitalization					
Uncomplicated delivery	8 (2.6%)	0 (0.00%)	8 (2.6%)	0.517	0.97
Diabetic ketoacidosis	8 (2.6%)	8 (2.6%)	16 (5.1%)		
Change in general condition	24 (7.70%)	6 (1.9%)	30 (9.6%)		
Stroke	24 (7.7%)	6 (1.9%)	30 (9.6%)		
Cesarean section	62 (19.9%)	14 (4.5%)	76 (24.4%)		
Coma	2 (0.6%)	2 (0.6%)	4 (1.3%)		
Gastroenteritis	6 (1.9%)	4 (1.3%)	10 (3.2%)		
Head trauma	26 (8.3%)	8 (2.6%)	34 (10.9%)		
Infectious syndrome	8 (2.6%)	8 (2.6%)	16 (5.1%)		
Postoperative monitoring	16 (5.1%)	10 (3.2%)	26 (8.3%)		
Acute retention of urine	12 (3.9%)	4 (1.3%)	16 (5.1%)		
Renal failure	8 (2.6%)	2 (0.6%)	10 (3.2%)		
Other conditions	22 (7.1%)	14 (4.5%)	36 (11.5%)		
Total	226 (72.4%)	86 (27.6%)	312 (100%)		

**Table 8:** Resistance profile (%) of enterobacteria and non-enterobacteria strains to the antibiotics used.

Antibiotics	AMX	CMA	TIC	TZP	CF	FOX	CTX	CAZ	ERT	IMI	AN	GEN	TOB	N/A	CIP	OFX	TIN	SXT
*Escherichia coli*	100	21.74	100	17.39	32.56	30.43	43.48	43.48	-	-	-	56.52	30.43	86.96	73.91	69.56	21.74	95.65
*Klebsiella* *pneumoniae*	100	40	100	30	80	30	80	80	-	30	10	80	20	70	50	50	30	70
*Enterobacter* *aerogenes*	25	75	100	-	75	75	50	-	-	-	-	50	50	75	50	50	25	100
*Citrobacter freundii*	-	100	50	-	100	100	-	-	-	-	-	50	50	100	50	50	-	50
*Pseudomonas* *lutola*	-	-	-	100	-	-	-	100	-	-	100	-	100	-	100	100	-	100
*Pseudomonas* *aeruginosa*	-	-	-	-	-	-	100	-	-	-	-	-	-	-	-	-	-	-
*Pseudomonas putida*	-	-	100	100	-	-	100	-	-	100	-	100	-	-	100	100	-	100
*Sphingomonas paucimobilis*	0	0	100	100	0	0	100	100	0	100	100	100	100	0	100	100	0	100

AMX: Amoxicillin; AMC: Amoxicillin/Clavulanic Acid; TIC: Ticarcillin; TZP: Piperacillin/tazobactam; CF: Cefalotin; FOX: Cefoxitin; CTX: Cefotaxime; CAZ: Ceftazidim; ERT: Ertapenem; IMI: Imipenem; AN: Amikacin; GEN: Gentamicin; TOB: Tobramycin; NA: Nalidixic acid; CIP: Ciprofloxacin; OFX: Ofloxacin; NIF: Nitrofurantoin; SXT: Trimethoprim/Sulfamethoxazole; -: No resistance.

**Table 9:** Percentages of resistance genes detected from bacterial species isolated from urine.

Isolated Species	Genes				
	*bla*_TEM_	*bla*_SHV_	*bla*_CTXM15_	*bla*_NDM_	*bla*_CTXM1_
*Escherichia coli*	20 (50%)	10 (25%)	6 (15%)	4 (10%)	12 (30%)
*Klebsiella pneumoniae*	10 (55.6%)	8 (44.4%)	4 (22.2%)	2 (11.1%)	6 (33.3%)
*Citrobacter freundii*	2 (100%)	-	2 (100%)	-	2 (100%)
*Pseudomonas putida*	2 (100%)	2 (100%)	2 (100%)	-	2 (100%)
*Sphingomonas* *paucimobilis*	-	-	-	-	-
*Enterobacter aerogenes*	2 (100%)	2 (100%)	-	-	-
**Total**	**36 (42.9%)**	**22 (26.2%)**	**14 (16.7%)**	**6** **(7.2%)**	**24** **(28.6%)**

## Data Availability

All data generated and/or analyzed during the current study are included in this published article. The datasets used and/or analyzed during this study are also available from the corresponding author on reasonable request.
